# Elevated [11C]-D-Deprenyl Uptake in Chronic Whiplash Associated Disorder Suggests Persistent Musculoskeletal Inflammation

**DOI:** 10.1371/journal.pone.0019182

**Published:** 2011-04-19

**Authors:** Clas Linnman, Lieuwe Appel, Mats Fredrikson, Torsten Gordh, Anne Söderlund, Bengt Långström, Henry Engler

**Affiliations:** 1 Department of Psychology, Uppsala University, Uppsala, Sweden; 2 McLean Hospital, Harvard Medical School, Boston, Massachusetts, United States of America; 3 The PET Center in Uppsala, Uppsala, Sweden; 4 Laboratory of Pain Research, Division of Anesthesiology and Intensive Care Medicine, Department of Surgical Sciences, Uppsala University Hospital, Uppsala, Sweden; 5 Department of Physiotherapy, School of Health, Care and Social Welfare, Mälardalen University, Västerås, Sweden; 6 Section of Physiotherapy, Department of Neuroscience, Uppsala University, Uppsala, Sweden; 7 Department of Biochemistry and Organic Chemistry, Uppsala University, Uppsala, Sweden; 8 Neuropsychopharmacology Section, Faculty of Medicine, Imperial College, London, United Kingdom; 9 Centre of Nuclear Research and Faculty of Medicine, University of the Republic, Montevideo, Uruguay; 10 Uruguayan Centre of Molecular Imaging, Montevideo, Uruguay; 11 Department of Nuclear Medicine, Uppsala University Hospital, Uppsala, Sweden; Charité Universitaetsmedizin Berlin, Germany

## Abstract

There are few diagnostic tools for chronic musculoskeletal pain as structural imaging methods seldom reveal pathological alterations. This is especially true for Whiplash Associated Disorder, for which physical signs of persistent injuries to the neck have yet to be established. Here, we sought to visualize inflammatory processes in the neck region by means Positron Emission Tomography using the tracer ^11^C-D-deprenyl, a potential marker for inflammation. Twenty-two patients with enduring pain after a rear impact car accident (Whiplash Associated Disorder grade II) and 14 healthy controls were investigated. Patients displayed significantly elevated tracer uptake in the neck, particularly in regions around the spineous process of the second cervical vertebra. This suggests that whiplash patients have signs of local persistent peripheral tissue inflammation, which may potentially serve as a diagnostic biomarker. The present investigation demonstrates that painful processes in the periphery can be objectively visualized and quantified with PET and that ^11^C-D-deprenyl is a promising tracer for these purposes.

## Introduction

Chronic musculoskeletal pain syndromes are common, cause extensive individual suffering and place a large burden on health care in society. Yet, pain remains notoriously difficult to visualize and diagnose objectively. Furthermore the pathophysiology of persistent pain is elusive and there is a great need for ways to visualize and quantify pain mechanisms. One common chronic pain syndrome is Whiplash Associated Disorder (WAD), where the onset is a soft tissue sprain of the neck caused by a rear end motor vehicle collision or a similar trauma. The injury is usually benign [1] and resolves within a couple of months, but in a sub-portion of the population, whiplash injuries proceed to chronic debilitating pain. Generally accepted diagnostic criteria for WAD exist [2], but the etiology of chronic symptoms is not known [3], and there is a lack of an objective biomarker for chronic WAD [4]. Recent magnetic resonance imaging (MRI) studies of the neck in WAD have indicated structural abnormalities in the deep cervical muscles and ligaments, typically around the uppermost vertebras [5], [6]. However, such findings can also be present in healthy subjects, and thus largely fail to distinguish between WAD and normal, pain-free natural variations [6]. Furthermore, structural imaging does not capture on-going biological processes; where as molecular imaging with positron emission tomography (PET) has the potential to visualize such mechanisms.

One candidate PET tracer for musculoskeletal injuries and inflammation is C^11^ (S-(+)-(d)-D-deprenyl, or DDE). High DDE retention has been demonstrated in the synovium in arthritic knee joints [7]. This suggests that DDE can be used to visualize chronic inflammatory processes even though the specific binding mechanism of the tracer is not yet known. The present study explores if DDE retention is elevated in the neck region in chronic WAD patients as compared to pain free controls. We hypothesized that WAD patients would have an elevated DDE retention in deep neck muscle regions.

## Materials and Methods

### Participants

Twenty-two patients (female/male: 18/4, mean age ±SD 36±12 years) with chronic neck pain after car accident involving whiplash trauma were recruited through hospital patient registries. 12 of the patients experienced a rear end collision, 5 patients experienced combined lateral and frontal collisions. Detailed collision descriptions were not available from 5 patients. All patients were diagnosed with whiplash associated disorder (WAD) grade II [2] and had experienced persistent pain between 6 and 24 months post trauma. The average time (±S.D.) since injury was 15±7 months. Specific exclusion criteria for patients were loss of consciousness at the time of the car accident, neurological symptoms, WAD due to other accidents than by car, and pain not related to WAD. The patient group was largely identical to that described in our recent brain imaging studies [3], [4]. All patients reported neck pain, limited range of neck movement and frequent headaches.

Fourteen healthy control subjects were also investigated. Control group A consisted of eight healthy and pain free volunteers (all females, mean age 33±9 years) recruited through advertisements. Control group B consisted of six patients with acute musculoskeletal pain (sprained ankle), but otherwise healthy and without current or prior neck pain (female/male: 3/3, mean age 36±14 years). General exclusion criteria for patients and controls were psychiatric disorder, somatic disease, substance abuse and pregnancy. The study was approved by the local ethical review board (Regionala Etikprövningsnämnden i Uppsala, approval Ups 02-124) and the local radiation protection committee (Uppsala University Hospital, Sweden). All participants provided written informed consent before entering the study.

### Subjective ratings

Prior to the PET investigation, participants rated their pain intensity on a 0-100 visual analogue pain scale (VAS), and completed the state portion of the Spielberger State Trait Anxiety Inventory (STAI-s) [5]. Patients were also administered the Neck Disability Index (NDI) [6], [7] and a whiplash questionnaire [2], [8] with accident description, health prior to whiplash injury and current symptom ratings. Pain localization drawings were obtained form all patients, but they were not detailed enough to asses the exact location of the neck pain, All participants refrained from analgesics and anti-inflammatory drugs one to three days (depending on drug half-life), from tobacco, alcohol and caffeine 12 h, and from food 3 h prior to the PET investigation.

### PET investigation

Carbon-11 marked DDE was produced for each PET investigation at GE healthcare, Uppsala Imanet following published procedures [9]. WAD patients were examined with DDE using an ECAT EXACT HR+ PET scanner (Siemens/CTI, Knoxville, USA). The scanner enabled acquisition of 63 contiguous planes of data with a distance of 2.46 mm resulting in a total axial field of view of 15.5 cm. Subjects were supine on the scanner couch with the head and neck comfortably and gently fixated, and positioned with the scanner field of view originating approximately 2 cm above the orbitomeatal line, allowing for 3D dynamical PET data acquisition from the base of the occipital bone down to the shoulder region. A 10 min transmission scan was performed using three retractable Germanium-68 rotating line sources. Thereafter, the patients received an intravenous bolus injection of DDE in the arm, approximately 5 MBq/kg body weight (mean dose ±SD  = 330±62 MBq). Simultaneously, a 45 min dynamic emission scan (3D mode) was initiated comprising of twelve frames: 4×30 s, 3×60 s, 2×300 s, and 3×600 s. These emission scans were reconstructed with a filter back projection using a 4 mm Hanning filter, resulting in a spatial resolution of about 5 mm in the field of view.

During the course of the study, a new PET-CT (GE discovery ST, General Electric Medical Systems, Milwaukee, WI) was installed and control subjects were investigated on this scanner, allowing for fusion of PET and CT images giving higher confidence in anatomical definitions. As different scanners were used for patients and controls, three of the 22 WAD patients were first investigated in the PET scanner and then also reinvestigated in the PET-CT scanner in order to ensure reproducibility across the two scanners. The time between these two investigations was about one month.

Control group A (8 healthy, pain free volunteers) was investigated on the PET-CT scanner, which has 24 rings of bismuth germinate crystal detectors. This scanner enabled the acquisition of 47 contiguous planes of data with a distance of 3.27 mm, resulting in a total axial field of view of 15.7 cm [10]. Positioning of the subjects was the same as for the patients. The PET-CT investigation was initiated with a short CT scan (140kV; auto mA 10–80 mA; pitch 1.75; speed 35) for attenuation correction of the PET emission data. After this procedure, the DDE (mean dose ±SD = 371±12 MBq) was administered as an intravenous bolus in the arm of the subject. As for the patient group, a 45 min dynamic emission scan (3D mode) was initiated with the same frame sequence. PET data was reconstructed using the OSEM (Ordered Subset Expectation Maximization) algorithm with 2 iterations and 32 subsets and with a Gaussian postprocessing filter 5.14 mm in width. All PET data were corrected for decay, photon attenuation, scatter, random coincidences and dead time. Control group B (6 subjects with acute ankle sprain) was investigated by means of a single 10 minute static image collected over the cervical neck region approximately 50 min post injection, DDE dose (424±58 MBq). Image reconstruction and post processing was identical to control group A.

### Image analysis

Images were first analyzed visually and locations of elevated tracer retention were specified with guidance from anatomical atlases and CT images when available. To quantify DDE retention, a series of regions of interest (ROIs) were defined in each subject. ROIs of identical size and shape were delineated in (I) a sphere in the non glandular regions with the most elevated retention, (called peak ROI), (II) a sphere left or right of the spineous process of axis (henceforth called right C2 ROI and left C2 ROI), (III) a sphere in the spongeus bone of the cervical vertebra 2 through 6 (vertebra ROIs), and (IV) a larger prolate spheroid in muscle tissue region without visually evident elevated retention (muscle ROI), see table 1 for a summary of ROI definitions. The ROIs were positioned in the last acquisition frame of the PET data in order to reflect specific uptake rather than blood flow. For each ROI and each time point, the radioactivity concentrations was extracted and recalculated with respect to the administered dose of radioactivity and the subject's body weight to represent standardized uptake values (SUV) [11] using an image analysis program (Amide Medical Image Data Examiner version 0.9.1) [12]. Plots of mean DDE SUV over time for each ROI in each subject, called time activity curves (TAC) were generated for subjects where dynamical PET data was available (patients and control group A). To test for differences in ROI uptake between patients and controls, two sided students T-test were performed on the SUV values from the last acquisition frame, 45 to 50 minutes post tracer injection.

**Table 1:** Region of Interest (ROI) definitions.

ROI	Volume (mm^3^)	Shape	Position/definition
Peak	500	Sphere	Non glandular soft tissue with highest uptake
Right C2	4200	Sphere	Region right of spineous process of C2
Left C2	4200	Sphere	Region left of spineous process of C2
Vertebra	500	Sphere	Spongeous bone of C2 through C6
Muscle	6300	Prolate spheroid	Muscle region without high uptake

### Test reproducibility evaluation

Three patients were imaged with both the Siemens HR+ PET and the GE discovery PET-CT system. Reproducibility across scanner type and session was evaluated by extracting the SUV TAC data at the 12 time points for the above specified ROIs from each subject in both PET scans. In addition, two more peak ROIs were generated for the regions with the second and third highest tracer uptake in each patient. As a metric of test reproducibility, the Pearson product moment coefficient of determination was calculated for SUV values at all time point from the HR+ and the PET-CT system in each patient for each ROI. See Figure S1 for comparisons of PET and PET-CT.

## Results

**Figure 1:**
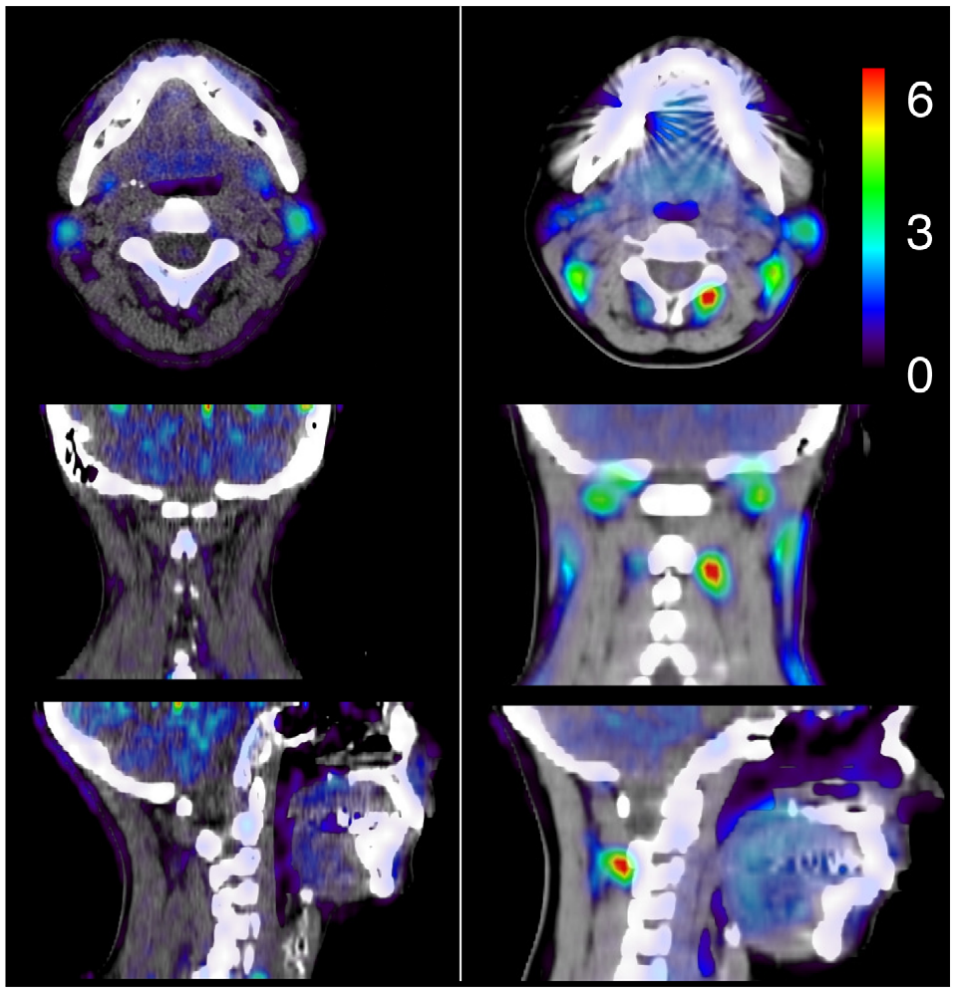
DDE uptake in a representative healthy control (left) and a whiplash patient (right). The patient displays high DDE uptake in the adipose tissue right of the spineous process of C2. PET images are overlaid on the subject's individual CT anatomy and tracer uptake is expressed as standardized uptake values.

Several soft tissue regions displayed visually evident elevated tracer retention in WAD patients, see figure 1. In both patients and controls, the uptake rate of DDE appeared stable 30 minutes post injection. Control group A and B did not differ significantly (p>0.3) on any of the ROI retention measures, therefore, to improve statistical power, ROI data from the two control groups was collapsed into one group for subsequent comparisons with the patient group.

**Figure 2:**
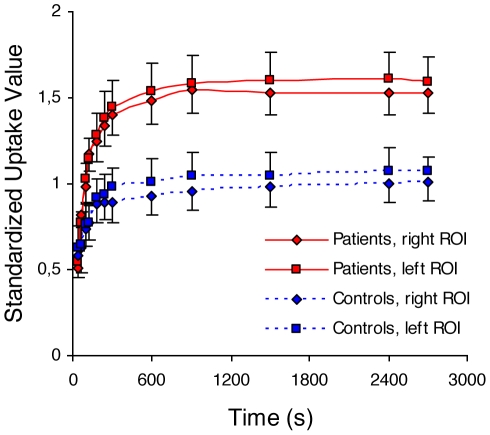
DDE uptake over time at the second cervical vertebra. Mean time activity curve indicated in standardized uptake values of DDE in two spherical regions of interest (ROI) left and right of the spineous process of C2. Bars indicate standard error. The area under the curve was significantly higher for patients (n = 22) than controls (n = 8) both in the right (p<0.004) and left (p<0.021) ROIs.

WAD patients had significantly higher DDE retention than controls in tissue regions adjacent to spineous process of the second vertebra (right C2 ROI p = 0.008, left C2 ROI p = 0.015), in the normal muscle tissue (p = 0.027) and in the peak soft tissue ROI (p = 0.035), but similar levels in the in the spongeus bone of the cervical vertebra (p = 0.48) see table 2 and figure 2.

**Table 2:** Standardizes Uptake Values (SUV) for DDE retention in regions of interest.

ROI	Patients SUV (±SD)	Controls SUV (±SD)	t-test p value
Peak	2.73±0.95	1.96±1.16	**0.035**
Right C2	1.52±0.55	1.05±0.41	**0.0082**
Left C2	1.59±0.69	1.08±0.42	**0.015**
Vertebra	2.22±0.52	2.09±0.56	0.48
Muscle	0.98±0.23	0.79±0.28	**0.027**

Peak retentions were located around the spineous process of C2 in most patients, and also at the insertion of rectus capitis posterior major in the occipital bone, see table 3. In the control group, retention in the fat between the sternocleidomastoid muscle and the levator scapulae muscle was the most common finding. In the three patients investigated with combined PET and CT, the location of the peak DDE retention was in the fat tissue surrounding the muscle, see figure 1.

**Table 3:** Non glandular soft tissue with the most elevated D-Deprenyl uptake in patients.

Subject	Location of Peak DDE retention	SUV
Patient 5	Left semispinalis cervicis muscle at C2	5.08
Patient 1	Left oblicus capitis inferior muscle	3.92
Patient 4	Right rectus capitis posterior major	3.81
Patient 10	Left insertion of rectus capitis posterior major in occipital bone	3.73
Patient 20	Left fat between sternocleidomatstoid and levator scapulae	3.66
Patient 12	Left semispinalis cervicis at C2	3.44
Patient 16	Nuchal ligament at axis	3.42
Patient 13	Left insertion of rectus capitis posterior major in occipital bone	3.38
Patient 22	Right fat between semispinalis capitis and C3	2.94
Patient 6	Left semispinalis cervicis muscle at C7	2.71
Patient 14	Left semispinalis cervicis at C2	2.49
Patient 21	Right fat at upper insertion of rectus capitis posterior major	2.32
Patient 8	Left levator scapulae at C4	2.13
Patient 2	Right oblicuus capitis inferior/semispinalis capitis at C2	2.07
Patient 7	Right multifidus muscle at C2	2.06
Patient 18	Right semispinalis at C6	2.06
Patient 3	Right rectus capitis posterior major	2.02
Patient 15	Splenius capitis muscle at C5	1.97
Patient 9	Left sternocleidomastoid muscle at C1	1.94
Patient 19	Left semispinalis at occipital insertion	1.90
Patient 11	Right insertion of sternocleidomastoideus in occipital bone	1.54
Patient 17	Right sternocleidomastoid muscle	1.36

SUV Standardized Uptake Value, anatomical locations are approximate, corresponding to the nearest identifiable region and may refer to surrounding tissue.

### Ratings

Patients reported significantly elevated VAS neck pain ratings (patients (n = 22) mean ±SD = 47±21, control group A (n = 8)  = 4±8, p<0.001). Control group B (n = 6) reported pain due to their acutely sprained ankle pain at rest (VAS = 23±20, p<0.05), but no neck pain. Patient's ratings of neck disabilities (NDI) were also significantly elevated (patients (n = 22) mean ±SD = 18±5, controls (n = 8)  = 2±1). No differences were found with respect to ratings of anxiety (STAI-s) at the time of investigation (patients (n = 22) mean ±SD  = 31±7, controls (n = 8)  = 30±5). Within the patient group, VAS pain ratings displayed a trend towards a positive correlation with DDE uptake in the C2 ROI (r = 0.39, p = 0.065).

### Test reproducibility across scanners

The test reproducibility between the two PET scanners used was high in all ROIs for the three subjects investigated on both scanners. The lowest observed coefficient of determination (r^2^) between the first and the second scan in SUV measures of the vertebra and muscle tissue was r^2^ ≥0.74 (p<0.001). In the presumably abnormal soft tissue peak ROIs, r^2^ was ≥0.63 (p<0.001). In the right and left C2 ROI's the lowest observed r^2^ was ≥0.93 (p<0.001) and r^2^ ≥0.86 (p<0.001) respectively. See also Figure S1.

### General tracer characteristics

**Figure 3:**
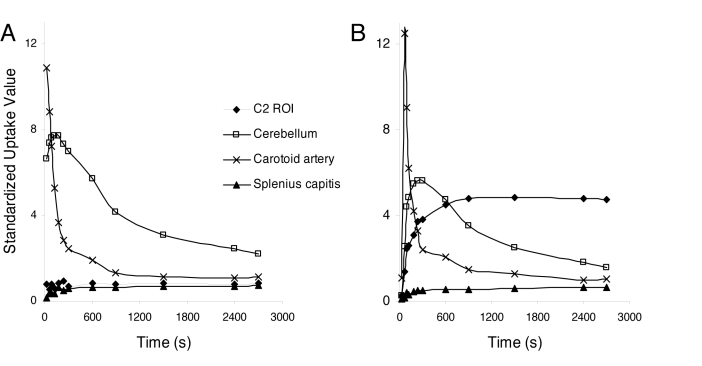
Tracer characteristics. Time activity curve in standardized uptake values are indicated for the C2 ROI, the cerebellum, the carotid artery and the splenius capitis muscle in **a**) a representative healthy controls, and **b**) a representative whiplash patient.

DDE displayed high retention in the submandibular and the parotid glands, and in the spongeous bone of the vertebra in all subjects. The external carotid artery region displayed high DDE retention during the early frames, but this decreased rapidly, characteristic of high blood flow without specific binding. In the cerebellum, the retention of the radioligand was moderate and non-specific. Muscle displayed generally low tracer retention (figure 3).

## Discussion

Patients displayed elevated DDE retention in cervical soft tissue, suggesting that localized chronic inflammation is apparent in around 50% of grade II whiplash associated disorder patients. From the PET-CT data, the site of tracer retention appeared to be localized to adipose tissue surrounding deep cervical muscles. The time activity curves (TAC) from the dynamic PET-data show a rapid increase in the first frames followed by a plateau in the last frames as with specific binding or trapping of the tracer. In regions with high blood flow, such as the carotid artery (figure 3), there was no retention of the tracer, suggesting that the elevated uptake in patients was not due to enhanced perfusion in affected areas. The trend towards a positive correlation between tracer retention and subjective pain ratings also suggests that DDE has may indicate the presence of painful inflammatory processes. Structural MRI studies indicate that WAD patients have high fatty infiltration in the cervical muscles [13], and an autopsy study suggest an increase in type IIC muscle fibers in the neck flexor muscles of patients with chronic neck pain [14]. Also, impairments in intramuscular microcirculation have been demonstrated in painful muscles [15]. Along with inflammation, these alterations in the microstructure and tissue composition of cervical tissue in WAD patients are possible mechanisms which could lead to elevated [^11^C]D-deprenyl retention. The tracer retention observed in fatty regions surrounding deep cervical muscle may indicate that adipose tissue is actively involved in the inflammatory process [16]. If DDE uptake can differentiate between traumatic and non-traumatic neck pain remains to be investigated.

The main limitation of this study is that we do not yet know the exact mechanism of the increased DDE uptake. (S-(+)-(d)-D-deprenyl is the mirror isomer of L-deprenyl, a well established MAO B inhibitor also known as selegeline [17]. However, the mirror isomer DDE is 20 to 500 times less potent as an MAO-B inhibitor than L-deprenyl [17]. L-deprenyl administration does not block DDE retention in inflammatory tissue, but corticosteroid injections does [18], suggesting that DDE may mark inflammation independent of MAO-B binding. There is some evidence suggesting that deprenyl has cytoprotective and antiapoptotic properties [19], [20], [21], effects that are possibly mediated either through an adaptive increase in superoxide dismutase activity [22], [23] or increased cell-cell adhesion [24]. Thus, further studies are needed to elucidate the exact retention mechanism of DDE and its role in inflammation. The present study is also limited by the use of two different PET scanners. However, the reproducibility across scanners was sufficient both normal tissue and regions with elevated tracer retention, supporting the validity of the observed group differences. Another possible limitation is that when [^18^F] fluorodeoxyglucose (FDG) PET is used to detect tumors, an uptake somewhat resembling the current finding has been observed in patients who are cold [25], [26]. The FDG uptake has been attributed to sympathetic noradrenergic outflow, leading to elevated metabolism in regions with brown adipose tissue. In the present study, all subjects fasted at least 3 h prior to the examinations, and were indoors in a centrally heated environment kept at 21°C for at least one hour prior to tracer administration. Both these procedures serve to reduce or eliminate brown fat metabolic activity [27].

This study represents a phase 1 diagnostic study [28] and further studies are needed before DDE PET can be recommended as a test in clinical practice [29]. Evaluating the specificity of the uptake against detailed clinical investigations of pain presentation, comparisons between DDE and other radioligands (i.e. FDG [30]), a more detailed anatomical resolution (through PET-CT or PET-MRI) and control groups including non-traumatic neck pain and headache patients are warranted.

### Conclusions

The present investigation demonstrates that painful processes in the periphery can be objectively visualized and quantified with PET and that DDE can be regarded as a promising tracer for these purposes. A large subset of patients with chronic pain after a whiplash injury displayed elevated DDE retention, suggestive of persistent peripheral tissue inflammation. The possibility to visualize and quantify sites of inflammation in chronic pain may be very useful in diagnosis and treatment monitoring. Inflammation imaging may also guide in examining and specifying mechanisms in debilitating chronic pain.

## Supporting Information

Figure S1 **Patient A, B and C scanned in both the Siemens HR+ PET (top row) and the GE Discovery PET-CT (bottom row).** Data is expressed as SUV values normalized for bodyweight and injected dose.
(TIF)
