# Supplementary figures and images for: Elevated [11C]-D-Deprenyl Uptake in Chronic Whiplash Associated Disorder Suggests Persistent Musculoskeletal Inflammation
Source: PLoS One. 2011 Apr 19;6(4):e19182. doi: 10.1371/journal.pone.0019182 (PMC3079741; doi:10.1371/journal.pone.0019182)

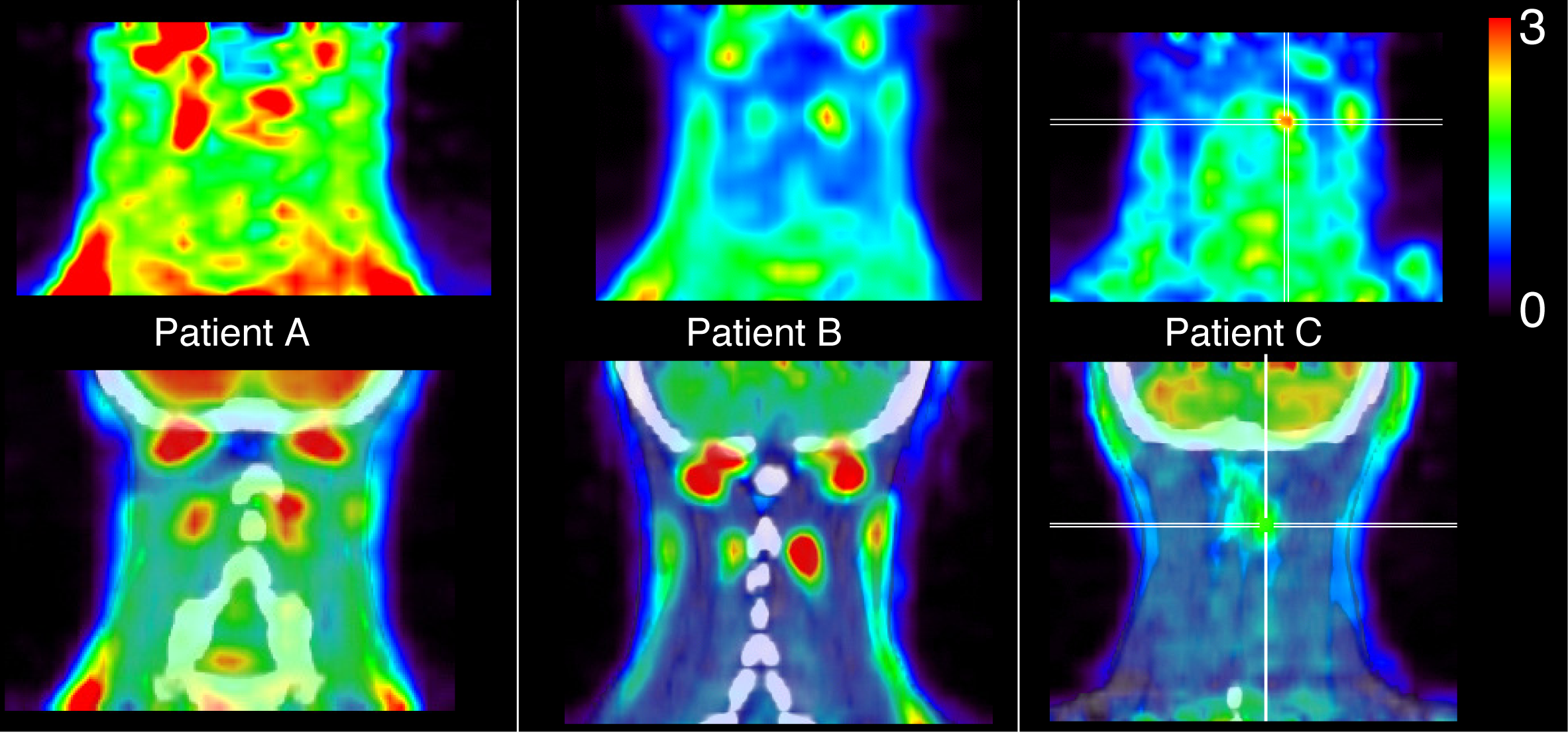

Supplement: Figure S1 — Patient A, B and C scanned in both the Siemens HR+ PET (top row) and the GE Discovery PET-CT (bottom row). Data is expressed as SUV values normalized for bodyweight and injected dose. (TIF) [file pone.0019182.s001.tif]
